# Prognostic Utility of FBLN2 Expression in Patients With Urothelial Carcinoma

**DOI:** 10.3389/fonc.2020.570340

**Published:** 2020-10-29

**Authors:** Wei-Ming Li, Ti-Chun Chan, Steven Kuan-Hua Huang, Wen-Jeng Wu, Hung-Lung Ke, Peir-In Liang, Yu-Ching Wei, Yow-Ling Shiue, Chien-Feng Li

**Affiliations:** ^1^Department of Urology, Kaohsiung Medical University Hospital, Kaohsiung, Taiwan; ^2^Department of Urology, School of Medicine, College of Medicine, Kaohsiung Medical University, Kaohsiung, Taiwan; ^3^Cohort Research Center, Kaohsiung Medical University, Kaohsiung, Taiwan; ^4^Department of Urology, Ministry of Health and Welfare Pingtung Hospital, Pingtung, Taiwan; ^5^Department of Medical Research, Chi-Mei Medical Center, Tainan, Taiwan; ^6^Division of Urology, Department of Surgery, Chi Mei Medical Center, Tainan, Taiwan; ^7^Regenerative Medicine and Cell Therapy Research Center, Kaohsiung Medical University, Kaohsiung, Taiwan; ^8^Institute of Medical Science and Technology, National Sun Yat-sen University, Kaohsiung, Taiwan; ^9^Department of Pathology, Kaohsiung Medical University Hospital, Kaohsiung, Taiwan; ^10^Department of Urology, School of Medicine, College of Medicine, Kaohsiung Medical University, Kaohsiung, Taiwan; ^11^Department of Pathology, Kaohsiung Municipal Ta-Tung Hospital, Kaohsiung, Taiwan; ^12^Institute of Biomedical Sciences, National Sun Yat-sen University, Kaohsiung, Taiwan; ^13^Department of Pathology, Chi-Mei Medical Center, Tainan, Taiwan; ^14^National Cancer Research Institute, National Health Research Institutes, Tainan, Taiwan

**Keywords:** *FBLN2*, bladder cancer, upper urinary tract cancer, urothelial carcinoma, prognosis

## Abstract

**Background:** Abnormal extracellular matrix (ECM) remodeling plays an essential role in urothelial carcinoma (UC) invasiveness and metastasis. Focusing on the ECM structural constituent (GO: 0005201), we recognized a significant upregulation of the fibulin 2 gene (*FBLN2*) during UC progression in a published UC transcriptome (GSE31684). Thus, we aimed to investigate the roles of *FBLN2* expression and its prognostic value in upper urinary tract UC (UTUC) and urinary bladder UC (UBUC) in our large, well-characterized cohort.

**Patients and Methods:** Clinicopathological data and formalin-fixed paraffin-embedded UC tissues were analyzed retrospectively. We determined *FBLN2* expression using immunohistochemical staining assessed by H-scores. *FBLN2* expression correlated with clinicopathological features and patient outcomes, including metastasis-free survival (MFS) and disease-specific survival (DSS). Statistical analyses were performed using Pearson's chi-square test, Kaplan–Meier estimates of DSS and MFS, and the Cox proportional hazards model. We used Ingenuity Pathway Analysis (IPA) to clarify the functional significance of dysregulated *FBLN2* in UC.

**Results:** Data from 295 UBUC and 340 UTUC patients were available for the final evaluation. Pearson's chi-square test showed that high *FBLN2* immunoexpression significantly correlated with adverse pathologic variables, such as advanced pathologic tumor stage, high histological grade, perineural invasion, vascular invasion, lymph node metastasis, and increased mitotic rate (all *p* < 0.05). Kaplan–Meier analysis demonstrated associations of high *FBLN2* expression with worse DSS (*p* < 0.001) and MFS (*p* < 0.001). Furthermore, multivariate analysis identified high *FBLN2* expression as an independent predictive risk factor for DSS [hazard ratio (HR) in UBUC, 2.306, *p* = 0.014; in UTUC, 2.561, *p* = 0.012] and MFS (HR in UBUC, 2.493, *p* = 0.001; in UTUC, 2.837, *p* = 0.001). IPA demonstrated that multiple signaling pathways were enriched, including the oxidative phosphorylation, mitochondrial dysfunction, and regulation of the epithelial–mesenchymal transition pathways.

**Conclusion:** High *FBLN2* expression was associated with adverse pathologic features and worse oncological outcomes and may serve as a prognostic biomarker for UC.

## Introduction

Urothelial carcinoma (UC) is a common urinary tract malignancy worldwide (1, 2). It may arise from the urinary bladder (UBUC) and upper urinary tract (UTUC), including the renal pelvis and ureter. Urinary bladder tumors account for 90–95% of UCs (1, 2). According to GLOBOCAN data, UBUC is the 10th most common cancer in the world, and its incidence is steadily rising (1). An estimated 550,000 new cases of UBUC were recorded worldwide in 2018 with ~200,000 deaths occurring during this same period (1). UTUCs are uncommon and account for only 5–10% of UCs in Western countries (1, 2).

For UBUC, ~75% of patients are initially diagnosed with non-muscle-invasive bladder cancer (NMIBC) while the remaining 25% are diagnosed with muscle-invasive bladder cancer (MIBC) (3, 4). Transurethral resection of the bladder tumor followed by intravesical instillation therapy constitutes the standard treatment for NMIBC (3). Different treatment modalities are suggested for MIBC, including radical cystectomy, bladder-preserving approaches, neoadjuvant or adjuvant therapy, and systemic therapy for advanced disease (4). For UTUC, approximately two-thirds of patients have invasive tumors at diagnosis. Although kidney-sparing surgery is recommended for low-risk UTUC without compromising oncological outcomes, radical nephroureterectomy with bladder cuff excision is still the standard treatment for most UTUCs (5). Despite advancements in surgical techniques and drug discovery, the prognosis for UC has not significantly improved over the past two decades (3–5).

UC tumor heterogeneity occurs at multiple levels and directly affects clinical care and treatment efficacy (6). Patients with UC typically die from tumor recurrence and metastasis (3–5). Consequently, a better understanding of the UC aggressiveness mechanism is crucial for improving patient prognosis. The hallmarks of cancer comprise six biological capabilities, including activating invasion and metastasis (7). Abnormal extracellular matrix (ECM) is a major component of a tumorigenic microenvironment as well as pre-metastatic and metastatic niches (8, 9). The process of cancer invasion and metastasis involves multifaceted molecular mechanisms in the cell and matrix interactions, which are reflected in variable up- and downregulation of multiple molecules (8, 9).

To identify transcripts that are potentially involved in UC invasiveness and metastasis, we performed data mining and processing of a public transcriptome dataset, focusing on the ECM structural constituent. We found that high transcript expression of fibulin 2 (*FBLN2*) was significantly associated with advanced pathologic stage and tumor metastasis in UBUC, suggesting its role in cancer progression.

The human *FBLN2* gene, mapped to chromosome 3p25.1, encodes a protein that is a member of the fibulin family, which is characterized by repeated epidermal growth factor (EGF)-like domains and a unique C-terminal fibulin-type module (10). Members of this family are associated with basement membranes and elastic ECM fibers. They play a central role in matrix stabilization by forming homodimeric complexes (11–13). *FBLN2* acts as a matrix organizer in the ECM to maintain tissue architecture. It is also highly expressed during organ development, including in skeletal, heart, and neuronal structures (11–13). Recently, an increasing number of studies have highlighted the involvement of *FBLN2* in carcinogenesis (14). In lung and pancreatic cancers, it exhibits a pro-tumor effect (15–17). However, *FBLN2* is a tumor suppressor in breast cancer and nasopharyngeal carcinoma (NPC) (18–20). Until now, the potential significance of *FBLN2* in UC has not been systematically analyzed. Consequently, in the present study, we intended to evaluate *FBLN2* expression and prognostic utility in our well-characterized UTUC and UBUC cohorts.

## Patients and Methods

### Data Mining of the Gene Expression Omnibus (GEO) Dataset

A transcriptome dataset (GSE31684), including 93 UBUC patients who received radical surgery, was collected from the NCBI GEO database. To quantify the gene expression level, we analyzed all probe sets without preselection or filtering and imported the raw files into Nexus Expression 3 software (BioDiscovery, El Segundo, CA, USA). Under supervision, comparative analyses were conducted to discover the significantly differentially expressed genes related to the ECM structural constituent (GO:0005201) by comparing tumor stage (high stage vs. low stage) and metastatic events (metastasis vs. non-metastasis). We chose differentially expressed genes (*p* < 0.01 and log_2_ ratio >0.3) for further study. From the Oncomine database, we selected two studies, Sanchez-Carbayo et al. (21) and Blaveri et al. (22), to evaluate the expression profiles of *FBLN2* in UBUC.

### Study Population

The study cohort consisted of 340 UTUC and 295 UBUC patients who received surgery with curative intent at a single medical center between 1998 and 2004. None of them received preoperative radiotherapy or chemotherapy. Clinical and pathologic characteristics as well as outcome data were retrospectively collected from patient medical charts. Histological grading was based on the World Health Organization criteria (23) while tumor staging was determined according to the American Joint Committee on Cancer system (24). The study was approved by the Institutional Review Board (IRB10501-005).

### Immunohistochemistry and Scoring

Four-micrometer-thick sections from formalin-fixed, paraffin-embedded blocks were sectioned on pre-coated slides followed by deparaffinization, rehydration, and antigen retrieval as previously described (25, 26). Next, we blocked endogenous peroxidase using 3% hydrogen peroxide solution. The sections were then incubated with the primary antibody against *FBLN2* (1:200, Catalog Number biorbyt-orb69091) for 1 h. Primary antibodies were detected using a ChemMate EnVision^TM^ Detection kit (DAKO, Carpinteria, CA). Sections processed without the primary anti-*FBLN2* antibody were used as negative controls. As for positive controls, we included HT 1197 cell block sections known to express high *FBLN2*. Two independent pathologists quantified *FBLN2* protein expression levels by integrating the percentage and intensity of immunostaining in the cytoplasm of UC cells to generate the H-score using the formula ΣPi(i +1), in which Pi represents the percentage of stained tumor cells (0 to 100%) and i is the intensity of immunoreactivity (0 to 3+). The immunoreactivity was divided into low and high expression levels based on the median H-score.

### Functional Interpretation of The Cancer Genome Atlas (TCGA) Data and Network Analysis

The RNA sequencing and clinical data were downloaded from the TCGA BLCA (Bladder Urothelial Carcinoma) database through the c-BioPortal platform. To understand the functional significance of dysregulated *FBLN2* in UC, we used Ingenuity Pathway Analysis (IPA; http://www.ingenuity.com) to analyze ingenuity canonical pathways, upstream regulators, diseases and biological functions, and gene networks. These core analyses were used to interpret the common differentially expressed genes between high *FBLN2*-expressing and low *FBLN2*-expressing UCs.

### Statistical Analysis

The association between *FBLN2* expression and various clinicopathological features was assessed using Pearson's chi-square test. We analyzed two end points, including metastasis-free survival (MFS) and disease-specific survival (DSS). Univariate and multivariate analyses were used to identify relevant *FBLN2* expression and clinicopathological characteristics among the predictors of DSS, measured from curative surgery to the time of cancer death, and MFS, measured from curative surgery to the first metastasis. Survival curves were drawn using the Kaplan–Meier method with a log-rank test. All significant parameters for univariate analysis were included in the multivariate Cox proportional hazards model to identify the independent predictors. SPSS Statistics V.17.0 software (IBM, Armonk, NY, USA) was utilized for statistical analyses. Statistical significance was set at *p* < 0.05.

## Results

### Association Between *FBLN2* Gene Expression and the ECM Structural Constituent in the UBUC Transcriptome

**Figure 1:**
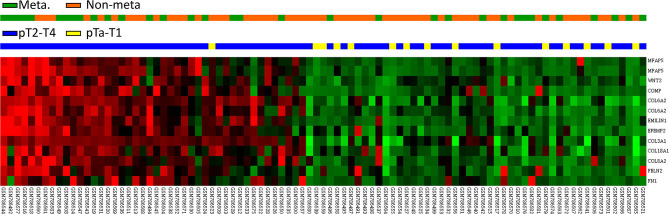
Expression profiles of genes associated with the extracellular matrix structural constituent (GO:0005201) extracted from a published transcriptome of urothelial carcinoma (GSE31684) in the Gene Expression Omnibus database. *FBLN2* was found to be one of the most significantly upregulated genes.

**Figure 2:**
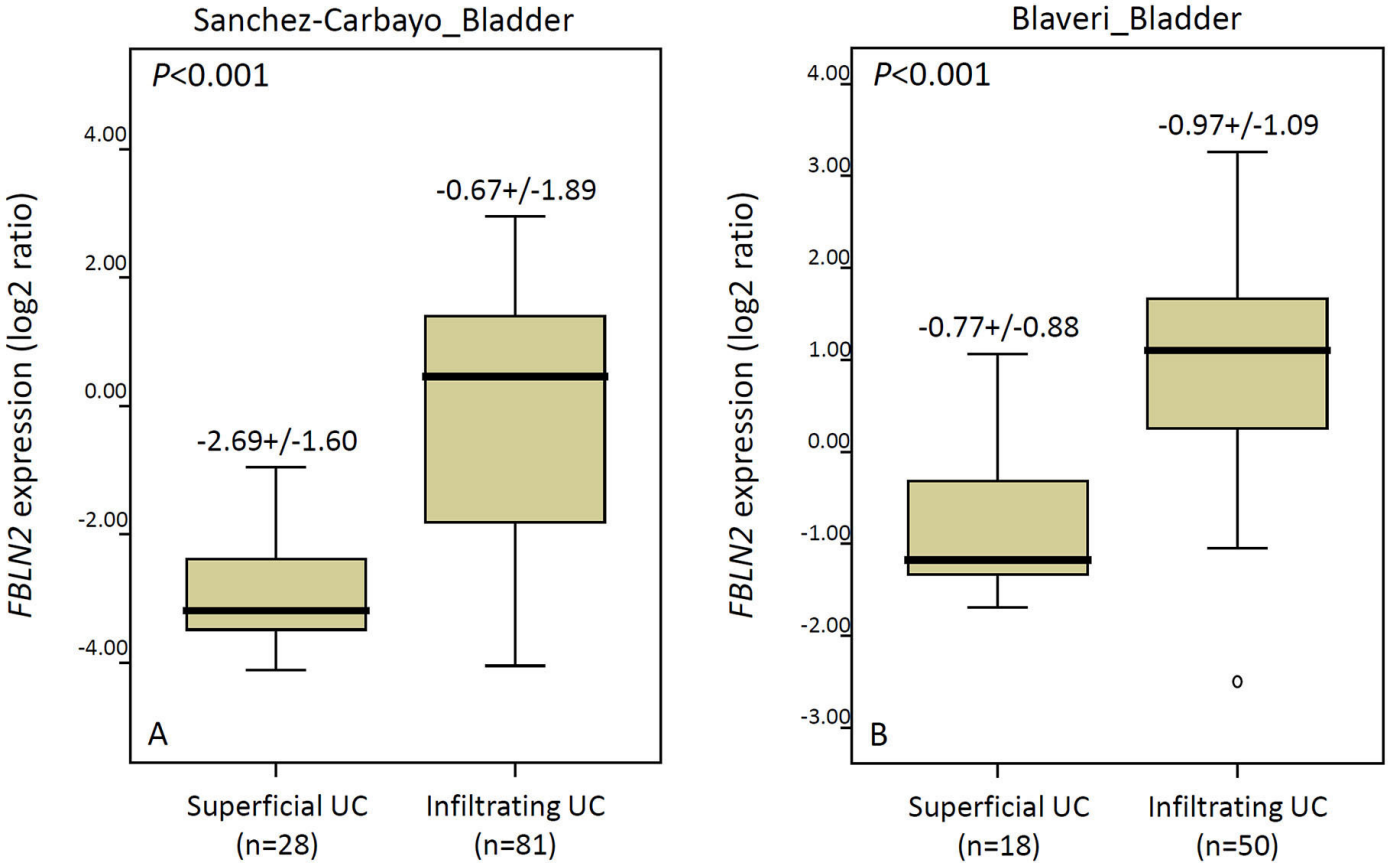
The Oncomine database shows significantly upregulated *FBLN2* in infiltrating urothelial carcinoma. The expression of *FBLN2* is demonstrated in two datasets (Sanchez-Carbayo and Blaveri) acquired from the Oncomine database.

We utilized a published UBUC transcriptome dataset (GSE31684) for data mining, including 93 patients undergoing radical cystectomy. There were 78 patients with muscle-invasive disease and 28 patients with lymph node metastasis. Through transcriptomic profiling, we identified 13 probes covering 11 transcripts associated with the ECM structural constituent (GO: 0005201). These genes were significantly upregulated in the high pathologic stage and lymph node metastasis (Table 1 and Figure 1). We selected *FBLN2* for further study, as the role of *FBLN2* in cancer development is not straightforward; it may inhibit or promote tumorigenesis. Its function in UC is not well-elucidated. To further confirm the initial observations, we examined the Oncomine database for *FBLN2* expression in bladder cancers. The Oncomine database revealed that there was a significantly higher expression of *FBLN2* in infiltrating UBUC compared to superficial tumors (Figure 2, Supplementary Figures 1, 2). These findings encouraged us to elucidate the role of *FBLN2* in our well-characterized UTUC and UBUC cohorts.

**Table 1:** Summary of differentially expressed genes associated with extracellular matrix structural constituent (GO:0005201) and showed positive associations to cancer invasiveness and metastasis in the transcriptome of urothelial carcinoma of urinary bladder (GSE31684).

**Probe**	**Comparing T2-4 to Ta-T1**		**Comparing meta. to non-meta**.^****#****^		**Gene symbol**	**Gene title**	**Molecular function**
	**Log ratio**	***p*-value**	**log ratio**	***p*-value**			
203886_s_at	0.8918	0.0003	0.6266	0.0009	*FBLN2*	Fibulin 2	Calcium ion binding, extracellular matrix structural constituent, protein binding
204163_at	1.1047	<0.0001	0.6313	0.0001	*EMILIN1*	Elastin microfibril interfacer 1	Extracellular matrix constituent conferring elasticity, extracellular matrix structural constituent, identical protein binding, protein binding
205648_at	0.6169	0.0014	0.4056	0.0061	*WNT2*	Wingless-type MMTV integration site family member 2	Extracellular matrix structural constituent, signal transducer activity
205713_s_at	1.8386	<0.0001	1.1823	0.0001	*COMP*	Cartilage oligomeric matrix protein	Calcium ion binding, extracellular matrix structural constituent, protein binding
209082_s_at	0.6536	0.0015	0.4516	0.0042	*COL18A1*	Collagen; type XVIII; alpha 1	Extracellular matrix structural constituent, metal ion binding, protein binding, structural molecule activity, zinc ion binding
209156_s_at	2.1304	<0.0001	0.9732	0.0021	*COL6A2*	Collagen; type VI; alpha 2	Extracellular matrix structural constituent, protein binding, protein binding; bridging, structural molecule activity
209356_x_at	0.5664	0.0027	0.4818	0.0008	*EFEMP2*	EGF-containing fibulin-like extracellular matrix protein 2	Calcium ion binding, extracellular matrix structural constituent, protein binding, transmembrane receptor activity
209758_s_at	1.4195	<0.0001	0.5778	0.0087	*MFAP5*	Microfibrillar associated protein 5	Extracellular matrix structural constituent
213290_at	0.832	0.0002	0.5142	0.004	*COL6A2*	Collagen; type VI; alpha 2	Extracellular matrix structural constituent, protein binding, protein binding; bridging, structural molecule activity
213764_s_at	1.8425	<0.0001	0.8102	0.0034	*MFAP5*	Microfibrillar associated protein 5	Extracellular matrix structural constituent
214702_at	0.8698	<0.0001	0.3948	0.0100	*FN1*	Fibronectin 1	Collagen binding, extracellular matrix structural constituent, heparin binding, protein binding
215076_s_at	1.4494	<0.0001	0.5177	0.0031	*COL3A1*	Collagen; type III; alpha 1 (Ehlers-Danlos syndrome type IV; autosomal dominant)	Extracellular matrix structural constituent, structural molecule activity
221900_at	0.8558	<0.0001	0.3936	0.0014	*COL8A2*	Collagen; type VIII; alpha 2	Extracellular matrix structural constituent, protein binding, protein binding; bridging, structural molecule activity

# *Meta., distal metastasis developed during follow-up; Non-Meta.: no metastatic event developed*.

### Clinicopathological Features of our UC Cohorts

We included 340 UTUC and 295 UBUC patients with a mean age of 65.8 years (Table 2). In the UTUC cohort, 49 patients (14.4%) had coexisting ureteral and renal pelvic tumors, and 62 patients (18.2%) had multiple tumors. There were 159 patients (46.8%) with advanced stage (pT2-4) UTUC, 284 (83.5%) with high histological grade tumors, and 28 (8.2%) with metastatic lymph nodes at diagnosis. Vascular invasion (VI) and perineural invasion (PNI) were noted in 106 (31.2%) and 19 cases (5.9%), respectively. Further, 167 lesions (49.1%) had high mitotic activity.

**Table 2:** Correlations between *FBLN2* expression and other important clinicopathological parameters in urothelial carcinomas.

**Parameter**	**Category**	**Upper urinary tract urothelial carcinoma**				**Urinary bladder urothelial carcinoma**			
		**Case No**.	***FBLN2*** **expression**		***p*-value**	**Case No**.	***FBLN2*** **expression**		***p*-value**
			**Low**	**High**			**Low**	**High**	
Gender	Male	158	77	81	0.664	216	107	109	0.868
	Female	182	93	89		79	40	39	
Age (years)	<65	138	73	65	0.377	121	55	66	0.210
	≥65	202	97	105		174	92	82	
Tumor location	Renal pelvis	141	64	77	0.228	–	–	–	–
	Ureter	150	77	73		–	–	–	–
	Renal pelvis and ureter	49	29	20		–	–	–	–
Multifocality	Single	278	135	143	0.261	–	–	–	–
	Multifocal	62	35	27		–	–	–	–
Primary tumor (T)	Ta	89	63	26	<0.001^*^	84	62	22	<0.001^*^
	T1	92	57	35		88	44	44	
	T2–T4	159	50	109		123	41	82	
Nodal metastasis	Negative (N0)	312	165	147	<0.001^*^	266	141	125	0.001^*^
	Positive (N1–N2)	28	5	23		29	6	23	
Histological grade	Low grade	56	44	12	0.001^*^	56	38	18	0.003^*^
	High grade	284	126	158		239	109	130	
Vascular invasion	Absent	234	144	90	<0.001^*^	246	138	108	<0.001^*^
	Present	106	26	80		49	9	40	
Perineural invasion	Absent	321	168	153	<0.001^*^	275	144	131	0.001^*^
	Present	19	2	17		20	3	17	
Mitotic rate (per 10 high-power fields)	<10	173	104	69	<0.001^*^	139	85	54	<0.001^*^
	≥10	167	66	101		156	62	94	

* *Statistically significant*.

In the UBUC cohort, 123 patients (41.7%) had MIBC (pT2-4), and most patients (81%) had high histological grade tumors at initial diagnosis. Concerning lymph node status, 266 patients (92.2%) were N0 and 29 (7.8%) were N1–2. There were 156 lesions (52.9%) with high mitosis. Moreover, VI and PNI were observed in 20 (6.8%) and 49 tumors (16.6%), respectively.

### *FBLN2* Immunoexpression and Patient Characteristic Correlations in UC

**Figure 3:**
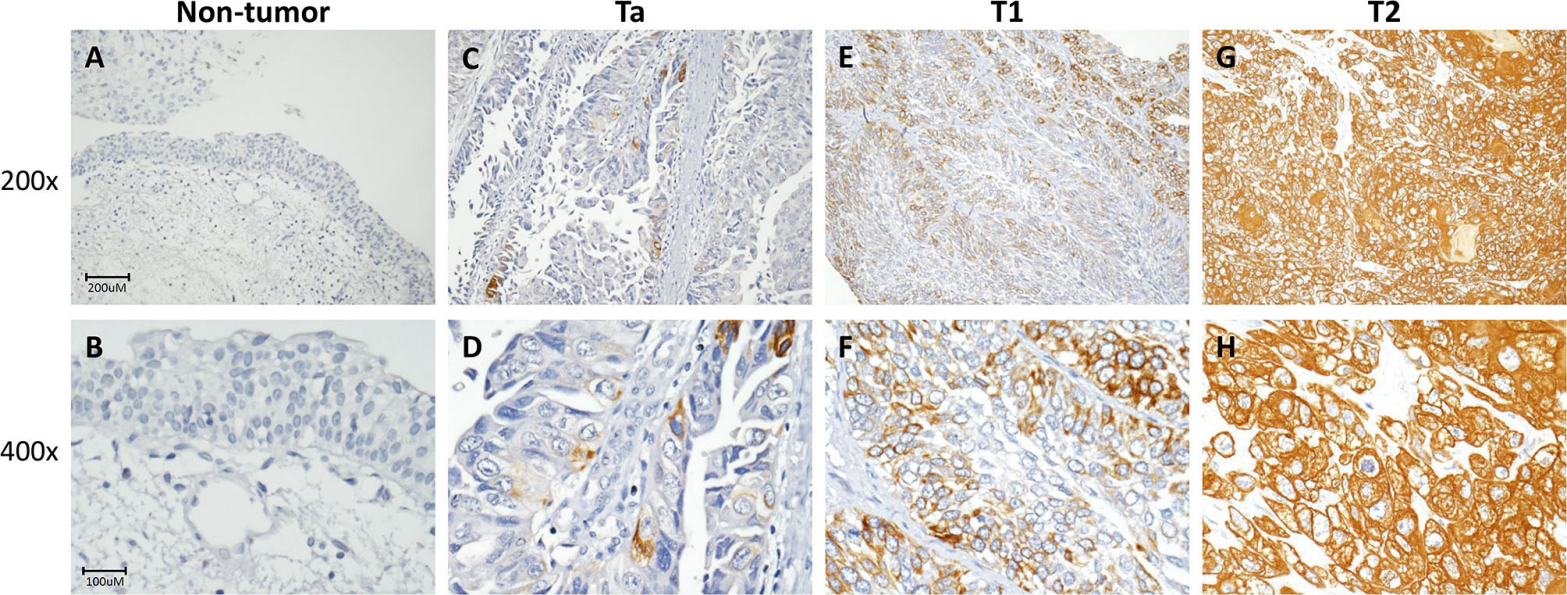
*FBLN2* immunohistochemistry. Representative sections of non-tumor **(A,B)**, non-invasive (Ta) **(C,D)**, superficial (T1) **(E,F)**, and muscle-invasive urothelial carcinoma (T2) **(G,H)** demonstrate stepwise increments in *FBLN2* expression (original magnification: 200× and 400×).

Immunohistochemical staining showed stepwise increments in *FBLN2* immunoreactivity from low stage UC to high stage UC (Figure 3). Table 2 shows the association of *FBLN2* immunoexpression with clinicopathological characteristics. In UTUC patients, high *FBLN2* expression was significantly associated with advanced pathologic tumor stage, high histological grade, lymph node metastasis, VI, PNI, and high mitotic activity (all *p* < 0.001). Similarly, in UBUC patients, we found significant associations between high *FBLN2* expression and advanced pT status (*p* < 0.001), high histological tumor grade (*p* = 0.003), lymph node metastasis (*p* = 0.001), VI (*p* < 0.001), PNI (*p* = 0.001), and high mitotic rate (*p* < 0.001).

### Prognostic Significance of *FBLN2* Expression

There were 113 patients, including 61 UTUCs and 52 UBUCs, who died of UC. In addition, 146 patients, including 70 UTUCs and 76 UBUCs, had subsequent metastatic tumor development. Univariate and multivariate analyses were performed to assess the prognostic impacts of *FBLN2* staining on cancer metastasis and patient death in UC.

**Figure 4:**
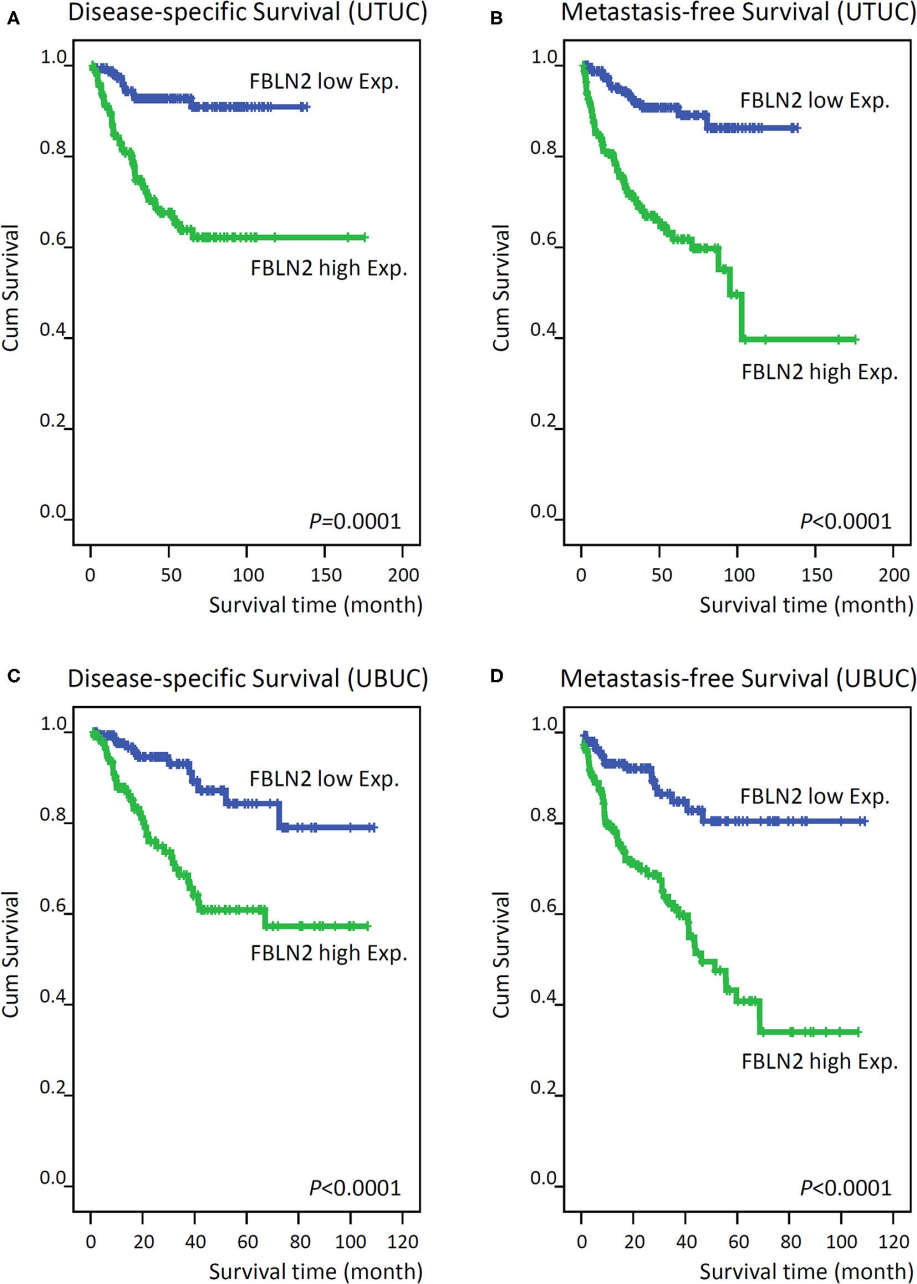
Kaplan–Meier plots show that *FBLN2* overexpression conferred significant prognostic effects on the disease-specific survival and metastasis-free survival of upper tract urothelial carcinoma (**A,B**, respectively) and urinary bladder urothelial carcinoma (**C,D**, respectively) patients.

In UTUC (Table 3), 29.4% of patients with high *FBLN2*-expressing tumors died of the disease, whereas only 6.5% of patients with low *FBLN2*-expressing tumors did. Furthermore, 32.9% of high *FBLN2*-expressing tumors developed metastasis, whereas only 8.2% of the tumors with low *FBLN2*-expressing tumors did. Kaplan–Meier analysis showed a significant association of high *FBLN2* expression with worse DSS (Figure 4A; *p* = 0.0001) and worse MFS (Figure 4B; *p* < 0.0001). Apart from *FBLN2* immunoexpression, we found that tumor location, multifocal tumors pathologic stage, lymph node metastasis, histological grade, PNI, and VI were significant prognosticators of MFS and DSS in the univariate analysis. Multivariate Cox regression analysis showed that *FBLN2* expression was independently predictive for DSS [hazard ratio (HR), 2.561; 95% confidence interval (CI), 1.219–4.968; *p* = 0.012] and MFS (HR, 2.837; 95% CI, 1.504–5.349; *p* = 0.001).

**Table 3:** Univariate log-rank and multivariate analyses for disease-specific and metastasis-free survivals in upper urinary tract urothelial carcinoma.

**Parameter**	**Category**	**Case No**.	**Disease-specific survival**					**Metastasis-free survival**				
			**Univariate analysis**		**Multivariate analysis**			**Univariate analysis**		**Multivariate analysis**		
			**No. of event**	***p*-value**	**RR**	**95% CI**	***p*-value**	**No. of event**	***p*-value**	**RR**	**95% CI**	***p*-value**
Gender	Male	158	28	0.8286	–	–	–	32	0.7904	–	–	–
	Female	182	33		–	–	–	38		–	–	–
Age (years)	<65	138	26	0.9943	–	–	–	30	0.8470	–	–	–
	≥65	202	35		–	–	–	40		–	–	–
Tumor side	Right	177	34	0.7366	–	–	–	38	0.3074	–	–	–
	Left	154	26		–	–	–	32		–	–	–
	Bilateral	9	1		–	–	–	0		–	–	–
Tumor location	Renal pelvis	141	24	0.0079^*^	1	–	0.476	31	0.0659	–	–	–
	Ureter	150	22		0.763	0.411–1.417		25		–	–	–
	Renal pelvis and ureter	49	15		1.675	0.462–6.067		14		–	–	–
Multifocality	Single	273	48	0.0026^*^	1	–	0.340	52	0.0127^*^	1	–	0.001^*^
	Multifocal	62	18		1.833	0.528–6.372		18		2.678	1.525–4.703	
Primary tumor (T)	Ta	89	2	<0.0001^*^	1	–	0.177	4	<0.0001^*^	1	–	0.118
	T1	92	9		3.575	0.763–16.756		15		3.178	1.034–9.772	
	T2–T4	159	50		4.226	0.926–19.284		51		2.251	0.704–7.192	
Nodal metastasis	Negative (N0)	312	42	<0.0001^*^	1	–	<0.001^*^	55	<0.0001^*^	1	–	0.001^*^
	Positive (N1–N2)	28	19		5.421	2.932–10.022		15		2.893	1.555–5.385	
Histological grade	Low grade	56	4	0.0215^*^	1	–	0.045^*^	3	0.0027^*^	1	–	0.158
	High grade	284	57		2.765	1.021–7.488		67		1.781	0.799–3.967	
Vascular invasion	Absent	234	24	<0.0001^*^	1	–	0.204	26	<0.0001^*^	1	–	0.002^*^
	Present	106	37		1.478	0.809–2.700		44		2.539	1.403–4.595	
Perineural invasion	Absent	321	50	<0.0001^*^	1	–	<0.001^*^	61	<0.0001^*^	1	–	0.027^*^
	Present	19	11		3.970	1.907–8.265		9		2.310	1.102–4.842	
Mitotic rate (per 10 high-power fields)	<10	173	27	0.167	–	–		30	0.0823	–	-	
*FBLN2* expression	≥10	167	34		–	–		40		–	–	
	Low	170	11	<0.0001^*^	1	–	0.012^*^	14	<0.0001^*^	1	–	0.001^*^
	High	170	50		2.561	1.219–4.968		56		2.837	1.504–5.349	

* *Statistically significant*.

In UBUC (Table 4) patients with high *FBLN2*-expressing tumors, 39.9% had tumor metastasis and 27.0% died of the disease, whereas only 11.6% of patients with low *FBLN2*-expressing tumors had subsequent metastatic tumors and 8.2% experienced cancer deaths. Following univariate analysis, we observed that advanced pT status, lymph node metastasis, high histological grade, VI, PNI, high mitotic activity, and high *FBLN2* expression (Figures 4C,D; all *p* < 0.0001) were associated with worse DSS and MFS. In the multivariate analysis, *FBLN2* expression status (HR, 2.306; 95% CI, 1.185–4.489; *p* = 0.014), pathologic stage, and mitotic activity were markedly associated with DSS. In addition, high *FBLN2* expression (HR, 2.493; 95% CI, 1.427–4.353; *p* = 0.001), high pathologic stage, and high mitotic activity significantly correlated with worse MFS.

**Table 4:** Univariate log-rank and multivariate analyses for disease-specific and metastasis-free survivals in urinary bladder urothelial carcinoma.

**Parameter**	**Category**	**Case No**.	**Disease-specific survival**					**Metastasis-free survival**				
			**Univariate analysis**		**Multivariate analysis**			**Univariate analysis**		**Multivariate analysis**		
			**No. of event**	***p*-value**	**RR**	**95% CI**	***p*-value**	**No. of event**	***p*-value**	**RR**	**95% CI**	***p*-value**
Gender	Male	216	41	0.4446	–	–	–	60	0.2720	–	–	–
	Female	79	11		–	–	–	16		–	–	–
Age (years)	<65	121	17	0.1136	–	–	–	31	0.6875	–	–	–
	≥65	174	35		–	–	–	45		–	–	–
Primary tumor (T)	Ta	84	1	<0.0001^*^	1	–	<0.001^*^	4	<0.0001^*^	1	–	0.006^*^
	T1	88	9		4.908	0.532–45.247		23		3.922	1.132–13.597	
	T2–T4	123	42		22.382	2.587–193.647		49		6.639	1.943–22.693	
Nodal metastasis	Negative (N0)	266	41	0.0002^*^	1	–	0.489	61	<0.0001^*^	1	–	0.060
	Positive (N1–N2)	29	11		1.283	0.634–2.598		15		1.816	0.975–3.381	
Histological grade	Low grade	56	2	0.0013^*^	1	–	0.911	5	0.0007^*^	1	–	0.907
	High grade	239	50		1.094	0.226–5.282		71		0.937	0.316–2.782	
Vascular invasion	Absent	246	37	0.0024^*^	1	–	0.121	54	0.0001^*^	1	–	0.845
	Present	49	15		0.581	0.293–1.155		22		0.941	0.513–1.727	
Perineural invasion	Absent	275	44	0.0001^*^	1	–	0.075	66	0.0007^*^	1	–	0.252
	Present	20	8		2.122	0.927–4.854		10		1.531	0.739–3.174	
Mitotic rate (per 10 high-power fields)	<10	139	12	<0.0001^*^	1	–	0.023^*^	23	<0.0001^*^	1	–	0.050^*^
*FBLN2* expression	≥ 10	156	40		2.190	1.115–4.305		53		1.677	0.999–2.815	
	Low	147	12	0.0001^*^	1	–	0.014^*^	17	<0.0001^*^	1	–	0.001^*^
	High	148	40		2.306	1.185–4.489		59		2.493	1.427–4.353	

* *Statistically significant*.

### Pathway, Functional Enrichment, and Interaction Network Analyses of *FBLN2*

The selection criteria of the differentially expressed genes included the top 200 most significant positively and negatively correlated genes to *FBLN2* expression in TCGA BLCA. Detailed information on the 400 differentially expressed genes is provided in Supplementary Tables 1,2. To further examine the associated gene signatures and functions of *FBLN2* in UC, these genes were analyzed by IPA. The data demonstrate the enrichment conditions of differentially expressed genes in canonical signaling pathways. Multiple signaling pathways were enriched, including oxidative phosphorylation, mitochondrial dysfunction, sirtuin signaling pathway, and regulation of the epithelial–mesenchymal transition pathway. The IPA results identified *CCR2, TGFB1*, and *TWIST1* among the top upstream regulators.

Additionally, in the disease and functional enrichment analyses, we identified that *FBLN2* may induce the alteration of numerous biological functions and diseases, such as cancer, organismal injury and abnormalities, connective tissue disorders, tissue development and cellular assembly and organization, and cellular function and maintenance. Subsequently, the differentially expressed genes were categorized into 28 networks. The top 3 networks had higher scores and more than 25 focused molecules. Genes encoding the collagen family proteins, including *COL10A1, COL14A1, COL16A1, COL1A2, COL3A1, COL5A1, COL5A2, COL6A1, COL6A2, COL6A3, COL8A1, COL8A2, OL1A2*, and *COL3A1*, along with 13 other dysregulated genes were involved in diseases and functions related to cancer, connective tissue disorders, and organismal injury and abnormalities, whereas 25 dysregulated genes, including *ADAM12, ADAMTS10*, and *ADAMTS2*, were involved in diseases and functions related to connective tissue disorders, hereditary disorders, and organismal injury and abnormalities. In addition, 9 nuclear genes encoding mammalian mitochondrial ribosomal proteins and 20 other dysregulated genes were involved in diseases and functions associated with metabolic diseases, organismal injury and abnormalities, and protein synthesis.

## Discussion

UC is a common and heterogeneous malignancy in the urinary system. Until recently, treatment for UC had seen little progress, and survival rates did not improve significantly over the last three decades. Therefore, the discovery of novel prognostic biomarkers remains an urgent concern in order to improve the clinical outcomes of UC patients. With advances in high-throughput technologies, many scholars have investigated the gene expression levels of UC samples and uploaded these data to public databases, such as GEO and TCGA (27, 28). These data offer chances for biomarker discovery, validation, and clinical application.

Using a public domain dataset (GSE31684), we demonstrated that *FBLN2* was a significantly upregulated gene related to the ECM structural constituent and associated with advanced tumor stage and metastatic disease in UBUC. The Oncomine database also revealed that infiltrating UBUC expressed significantly higher *FBLN2* than superficial tumors. Furthermore, the prognostic impact of *FBLN2* immunoexpression was validated in our well-characterized UC cohort. We demonstrated that *FBLN2* expression status was independently predictive for both MFS and DSS in UTUC and UBUC. In addition, high *FBLN2* expression was significantly associated with adverse pathologic tumor characteristics, such as advanced pathologic tumor stage, high histological tumor grade, lymph node metastasis, VI, PNI, and high mitotic rate. These results indicate that *FBLN2* plays a key role in UC development and progression. Thus, incorporation of *FBLN2* immunoexpression with standard pathologic and prognostic parameters may improve UC risk stratification.

Radical operation is the only curative therapy for UC. However, cancer metastasis and progression frequently occur over time, resulting in patient mortality (3–5). Deregulated ECM dynamics play important roles in cancer invasiveness and progression (29, 30). The ECM consists of a complex network of highly modified macromolecules, including fibrous proteins, proteoglycans, glycosaminoglycans, and glycoproteins, with different physical and biochemical properties (29, 30). It not only supports tissue architecture and integrity but also modulates and regulates cell functions such as adhesion, migration, proliferation, and differentiation (29, 30). Abnormal ECM dynamics result in cellular dysfunctions that promote cellular transformation and metastasis (29, 30). Fibulins, a family of glycoproteins, are related to elastic ECM fibers and basement membranes. They severed as a scaffold for the ECM (11–13). *FBLN2* can form homodimeric complexes and bind to many ECM components, such as integrin, fibronectin, and laminin, to stabilize the organization of ECM structures (10–13). Therefore, we selected *FBLN2* for further study.

Depending on the tissue context, *FBLN2* may act as either a tumor promoter or a tumor suppressor in different cancer models (15–20). For example, in lung cancer, Baird et al. demonstrated that *FBLN2* can drive malignant progression and promote tumor cell adherence to collagen and collagen cross-linking (15). They found that *FBLN2* was abundant in the ECM of human lung cancer. Knockdown of the *FBLN2* gene in lung cancer cells inhibited tumor growth and lymph node metastatic properties in a xenograft and orthotopic 129/SV mice model. However, in NPCs, Law et al. found that *FBLN2* was a tumor suppressor and had antiangiogenic effects (20). *FBLN2* was downregulated in NPCs but highly expressed in adjacent normal tissues. Exogenous expression of the *FBLN2* gene in NPCs suppressed cell proliferation, migration, invasion, and angiogenesis *in vitro*. *FBLN2* re-expression also inhibited tumor growth and angiogenesis *in vivo* (20). However, the roles of *FBLN2* in UC remain unclear.

In the present study, we demonstrated that high *FBLN2* immunoexpression was significantly associated with aggressive UTUC and UBUC characteristics, such as high tumor stage and grade, PNI, VI, lymph node metastasis, and high mitotic rate, suggesting important roles of *FBLN2* in UC progression and metastasis. These findings have some clinical applications. In NMIBC, it is critical to recognize the high-risk patients who will progress to MIBC (3, 4). *FBLN2* immunoexpression status may serve as a useful marker to classify these patients because high *FBLN2* expression is significantly related to muscle invasiveness and high-grade UBUC. For low-risk UTUC, kidney-sparing management may be a primary treatment without compromising oncological outcomes (5). However, in ureteroscopic biopsy tumors with high *FBLN2* expression, radical nephroureterectomy with bladder cuff excision should be suggested. High *FBLN2* immunoactivity was observed in high-grade, advanced-stage, and lymph node metastatic UTUC. For advanced or metastatic UC, cisplatin-based combination chemotherapy or checkpoint inhibitor immunotherapy has survival benefits (4, 5), suggesting the need for early identification of patients with progressive and metastatic potential. Here, we showed that UC patients with high *FBLN2* expression and aggressive pathologic features were more likely to develop distant metastasis. These findings may accelerate early recognition and aggressive UC management, such as early radical surgery, systemic chemotherapy, or immunotherapy.

Although the exact mechanism underlying the role of *FBLN2* in UC progression has not been well-studied, its expression may indicate a distinctive subtype in UC with aggressive characteristics and worse oncological prognosis. Several metabolic altered pathways are involved in UC tumorigenesis and progression, representing potential therapeutic targets (31). Using the IPA database, we identified pivotal canonical pathways according to the differential gene expressions. Among the predicted pathways, oxidative phosphorylation and mitochondrial dysfunction were the top pathways with a significant correlation, suggesting critical roles of *FBLN2* in metabolic changes and UC progression. Moreover, *FBLN2* was also significantly associated with the sirtuin signaling pathway, which is involved in epithelial to mesenchymal transition promotion and UC progression (32). Therefore, further studies are necessary to elucidate the exact molecular mechanisms underlying *FBLN2* effects on these pathways in UC.

There are some limitations in our research. First, this is a single-institution retrospective study. Second, the precise molecular mechanism resulting in disease progression and adverse outcomes in *FBLN2*-overexpressing UC has not been well-clarified. Third, no standardized immunostaining and scoring scheme for *FBLN2* expression is currently available. In this study, we followed the standard protocol of immunostaining and estimated both the intensity and percentage of positively stained UC cells to yield an H-score on account of its excellent correlation to immunoblots (33). In addition, two independent pathologists evaluated all slides to reduce discrepancy and come to a consensus for every case. Finally, prospective multicenter studies are necessary to verify our findings.

In conclusion, we demonstrated that high *FBLN2* immunoexpression is associated with aggressive tumor characteristics and independent prognostication of worse oncological outcomes in a large, well-characterized UC cohort. Thus, *FBLN2* may serve as a potential prognostic biomarker to identify high-risk UC patients for individualized therapy.

## Data Availability Statement

The datasets presented in this study can be found in online repositories. The names of the repository/repositories and accession number(s) can be found in the article/Supplementary Material.

## Ethics Statement

The studies involving human participants were reviewed and approved by the Institutional Review Board (IRB10501-005) of Chi Mei Medical Center. Written informed consent for participation was not required for this study in accordance with the national legislation and the institutional requirements.

## Author Contributions

W-ML, Y-LS, and C-FL: study concept and design. T-CC, SH, P-IL, and Y-CW: acquisition of data. H-LK and C-FL: analysis and interpretation of data. W-ML, Y-LS, and C-FL: drafting of the manuscript. Y-LS and C-FL: critical revision of the manuscript for important intellectual content. W-ML and C-FL: statistical analysis. All authors agreed to be accountable for the content of the work.

## Conflict of Interest

The authors declare that the research was conducted in the absence of any commercial or financial relationships that could be construed as a potential conflict of interest.

## Supplementary Material

Supplementary Figure 1 Oncomine database illustration of *FBLN2* expression in Sanchez-Carbayo Bladder 2.

Supplementary Figure 2 Oncomine database illustration of *FBLN2* expression in Blaveri Bladder 2.

Supplementary Table 1 IPA. Top 200 most significant positively correlated genes to *FBLN2* expression in bladder cancer.

Supplementary Table 2 IPA. Top 200 most significant negatively correlated genes to *FBLN2* expression in bladder cancer.
