# Supplementary figures and images for: Prognostic Utility of FBLN2 Expression in Patients With Urothelial Carcinoma
Source: Front Oncol. 2020 Oct 29;10:570340. doi: 10.3389/fonc.2020.570340 (PMC7659889; doi:10.3389/fonc.2020.570340)

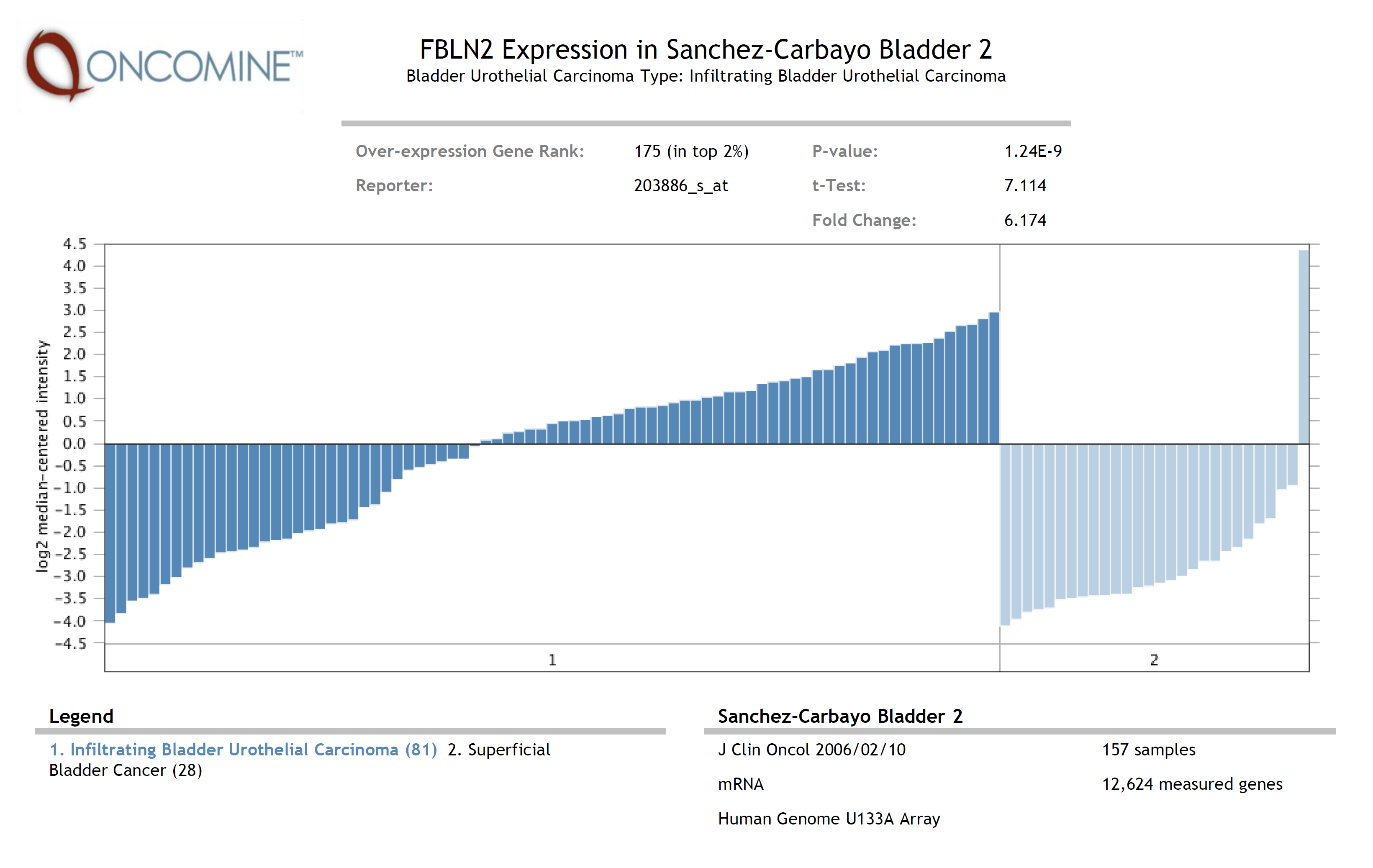

Supplement: Supplementary Figure 1 — Oncomine database illustration of FBLN2 expression in Sanchez-Carbayo Bladder 2. [file Image_1.TIF]

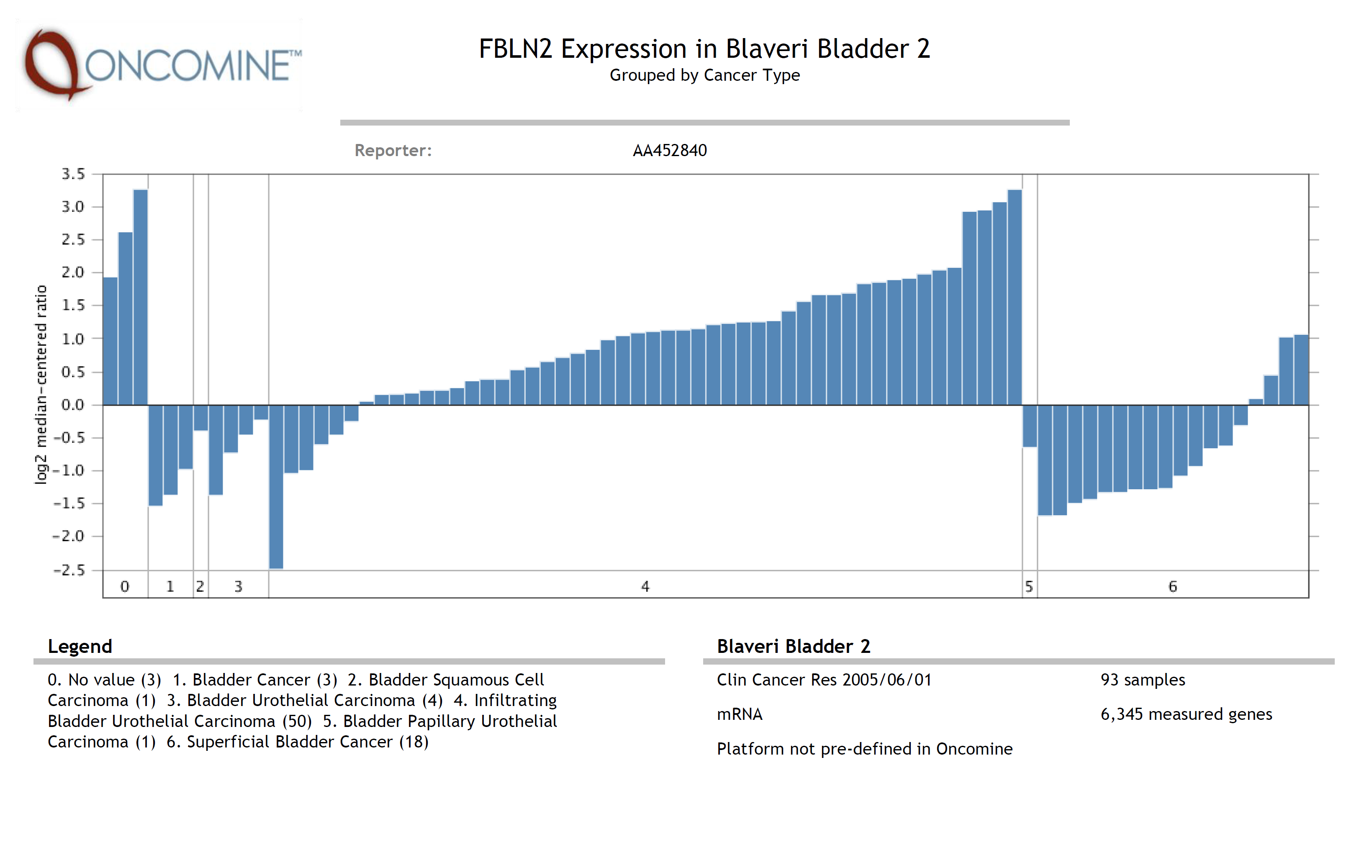

Supplement: Supplementary Figure 2 — Oncomine database illustration of FBLN2 expression in Blaveri Bladder 2. [file Image_2.TIF]
